# IgM mesangial deposition as a risk factor for relapses of adult-onset minimal change disease

**DOI:** 10.1186/s12882-021-02234-z

**Published:** 2021-01-12

**Authors:** Cheng-Wen Yang, Fan-Yu Chen, Fu-Pang Chang, Yang Ho, Bo-Sheng Wu, An-Hang Yang, Der-Cherng Tarng, Chih-Yu Yang

**Affiliations:** 1 Division of Nephrology, Department of Medicine, Far Eastern Memorial Hospital, Taipei, Taiwan; 2 Division of Nephrology, Department of Medicine, Taipei Veterans General Hospital, No. 201, Section 2, Shih-Pai Road, Beitou District, Taipei, 11217 Taiwan; 3 Faculty of Medicine, School of Medicine, National Yang-Ming University, Taipei, Taiwan; 4 Department of Pathology, Taipei Veterans General Hospital, Taipei, Taiwan; 5 Institute of Clinical Medicine, School of Medicine, National Yang-Ming University, Taipei, Taiwan; 6 Department and Institute of Physiology, National Yang-Ming University, Taipei, Taiwan; 7 Center for Intelligent Drug Systems and Smart Bio-devices (IDS2B), Hsinchu, Taiwan; 8 Stem Cell Research Center, National Yang-Ming University, Taipei, Taiwan

**Keywords:** Adult-onset, Minimal change disease, IgM mesangial deposition, Relapse

## Abstract

**Background:**

Immunoglobulin M (IgM) mesangial deposition in pediatric minimal change disease (MCD) has been reported to be associated with steroid dependence and poor renal outcomes. However, the evidence linking the impacts of IgM mesangial deposition to the treatment prognosis in adult-onset MCD is still elusive.

**Methods:**

In this retrospective cohort study, 37 adult patients with MCD received kidney biopsies from January 2010 to May 2020. Immunofluorescence microscopy was performed and the patients dichotomized according to IgM mesangial deposition (12 patients with positive IgM deposition; 25 patients with negative IgM deposition). We analyzed the clinical features, the dosage of immunosuppressive agents, and the response to treatment for 2 years between the two groups.

**Results:**

Analysis of the clinical symptoms, the dosage of immunosuppressive treatment, and the time to remission revealed no statistical difference between the groups. However, compared to the negative IgM group, the frequency of relapses was significantly higher in the positive IgM group during the two-year follow-up period (the negative IgM group 0.25 episodes/year; the positive IgM group 0.75 episodes/year, *p* = 0.029). Furthermore, multivariate linear regression revealed that the positivity of IgM mesangial deposition is independently associated with the frequency of relapses (regression coefficient B 0.450, 95% CI 0.116–0.784, *p* = 0.010).

**Conclusions:**

Our findings indicated that adult-onset MCD patients with IgM mesangial deposition have a high risk of relapses. Therefore, intensive monitoring of disease activity should be considered in MCD adults with IgM mesangial deposition.

**Supplementary Information:**

The online version contains supplementary material available at 10.1186/s12882-021-02234-z.

## Background

Minimal change disease (MCD) is the absence of glomerular changes, tubular injury, interstitial fibrosis, or sclerosis under light microscopy. The immunofluorescence (IF) analysis typically showed negative staining, but some reveal immunoglobulin M (IgM) positive [1]. IgM is a serum antibody and serves as a primary activator for the complement cascade. As a large molecule, IgM rarely diffuses and consequentially deposit in tissues [2]. The diffuse granular global mesangial IgM deposition may affect renal glomeruli, with similar effects to IgA nephropathy. Numerous pediatric MCD studies focused on the predictors of kidney function or treatment response with IgM deposition [3–5]. Such IgM deposition was observed in 11.9% of children and 4.3% of adults in a study [6].

A previous study indicated that MCD children with a positive IgM mesangial deposition were more likely to evolve into focal segmental glomerulosclerosis (FSGS) [7]. IgM deposition has been viewed as a transitional form between MCD and FSGS [8]. Deposition of IgM is also found in other subtypes of idiopathic nephrotic syndrome [4]. Children with idiopathic nephrotic syndrome and a positive IgM mesangial deposition were found to have reduced response to therapy [6].

A positive IgM mesangial deposition has been reported to be associated with steroid dependence and worse renal outcomes in the pediatric population [2, 5, 9]. On the other hand, previous studies only focused on the histopathology and natural history in adult-onset MCD with positive IgM mesangial deposition [4, 10, 11]. The clinical significance and prognosis of a positive IgM mesangial deposition in adult-onset MCD are still unknown. We hypothesized that a positive IgM mesangial deposition indicates a poor prognosis in adult MCD. We aimed to compare the treatment response in adult-onset MCD with or without IgM mesangial deposition. Our study included the time to partial remission, complete remission, time to the first relapse, and frequency of relapses.

## Methods

### Patients

**Fig. 1:**
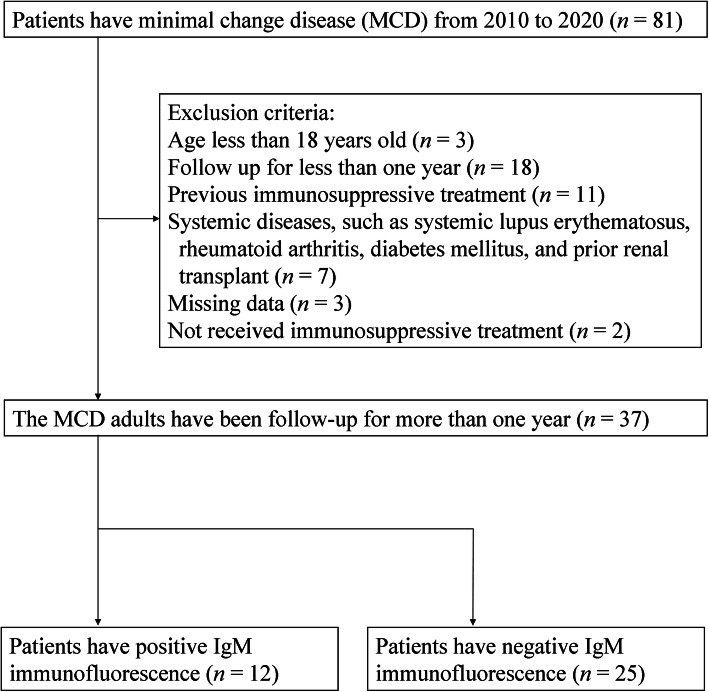
Flow diagram for the study

The protocol of this study was approved by the Institutional Review Board of Taipei Veterans General Hospital, Taipei, Taiwan. The protocol conformed with the ethical guidelines of the *Helsinki Declaration*. The need for informed consent was waived because of the retrospective nature of the study. We enrolled patients who were diagnosed as MCD by renal biopsy at the Taipei Veterans General Hospital from January 2010 to May 2020. Eighty-one samples were purely MCD, without significant tubulointerstitial, glomerular, and vascular lesions. As shown in Fig. 1, we excluded patients with age less than 18 years old, follow up for less than 1 year, previous immunosuppressive treatment, systemic diseases (such as systemic lupus erythematosus, rheumatoid arthritis, diabetes mellitus, and prior renal transplant), and missing data. All 37 patients had been examined for IgM mesangial deposition by IF microscopy.

### Study protocol and subjects

Nephrotic syndrome is characterized by overt proteinuria (more than 3.5 g/ 24 h), hypoalbuminemia (less than 2.5 g/dL), hyperlipidemia, and edema. Hematuria is defined by urinalysis (urine red blood cell (RBC) more than 3/high-power field (HPF)). We collected serial urine protein to creatinine ratio (UPCR), urine RBC, serum albumin, serum immunoglobulin, lipid profiles, blood pressure, and serum creatinine. The estimated glomerular filtration rate (eGFR) was estimated according to the Chronic Kidney Disease Epidemiology Collaboration formula [12]. IgM mesangial deposition (grade from 1+ to 3+) was categorized as the positive IgM group in our study. Partial remission (PR) was defined as the reduction of UPCR between 0.3 and 3.5 g/g with stable serum creatinine. Complete remission (CR) was defined as a UPCR of less than 0.3 g/g with normal serum creatinine. A relapse was defined as a UPCR of more than 3.5 g/g after CR, according to the Kidney Disease Improving Global Outcomes (KDIGO) guideline [13].

Immunosuppressive therapy was initiated after biopsy, and the majority of them used prednisolone. Adjuvant immunosuppressants included cyclosporine (CsA), mycophenolic acid (MPA), and cyclophosphamide (CYC). Because this was a retrospective study, the decision on dosages and types of immunosuppressive agents was at the discretion of each attending nephrologist.

### Statistical analysis

**Fig. 2:**
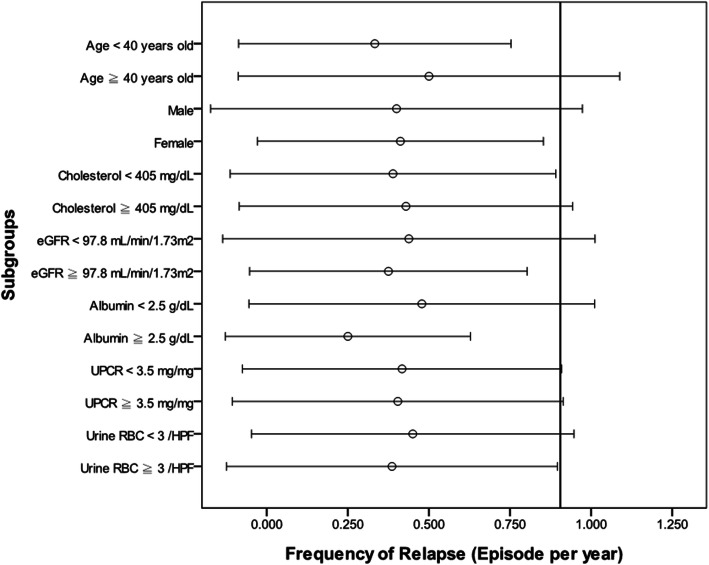
Estimated change in the frequency of relapses in subgroups. The reference line was the mean plus one standard deviation of the frequency of relapses for all patients, which is 0.905 episodes/year

Chi-square analysis or *Fisher*’s exact test was used for comparisons of categorical variables as appropriate. Continuous variables were compared by *Student*’s t-test. For linear regression analysis, the frequency of relapses was set as the dependent variable, and IgM-associated variables were used as independent variables. In subgroup analysis, patients were divided into two subgroups according to their mean value to examine the subgroup difference. The reference line in Fig. 2 was the mean value plus one standard deviation. SPSS version 19.0 for Windows (SPSS Inc., Chicago, Illinois, USA) was used for all statistical analyses. All probabilities were two-tailed, and a *p*-value of less than 0.05 was considered to be statistically significant.

## Results

### Baseline characteristics of the study subjects

Clinical characteristics of 37 patients with MCD are listed in Table 1. The average age of patients was 39.7 years old. There were no statistically significant differences in blood pressure, lipid profile, serum albumin, serum IgM, renal function, proteinuria, and hematuria between the two groups. There were no significant tubulointerstitial, glomerular, and vascular lesions by light microscopy. In immunohistology, IgM positivity was defined as IgM deposits in the mesangial area. The intensity of IgM was 1+ in five patients, 2+ in five patients, and 3+ in two patients. Among the positive IgM group, one patient also had positive IgG, two patients had positive complement 1q, and one patient had positive complement 3 (all in the positive IgM group). In patients of the negative IgM group, all IF examinations showed negative findings. Upon electron microscopic (EM) examination, all enrollees had the effacement of the podocyte foot processes. Only 3 out of 12 patients in the positive IgM group had mesangial deposition upon EM examination (*n* = 3; 25%). Because only a small amount of tissue was examined by EM, a negative finding of mesangial deposits on EM may just be a result of limited glomeruli being examined. The larger amount of glomeruli on IF may reduce sampling bias and the possibility of a false-negative result.

**Table 1:** Demographic characteristics and clinical features in adult-onset minimal change disease on light microscopy

Parameters	All	IgM (+)	IgM (−)	*p*-Value
Patient number (*n*)	37	12	25	
Age (year)	39.7 ± 16.5	38.5 ± 14.9	40.3 ± 17.6	0.755
Male gender (*n*; %)	16; 43.2	5; 41.7	11; 44.0	0.893
Body weight (kg)	65.6 ± 13.0	64.7 ± 11.5	66.0 ± 13.8	0.777
Blood pressure				
Systolic blood pressure (mmHg)	129.4 ± 23.6	134.9 ± 28.3	126.8 ± 21.1	0.332
Diastolic blood pressure (mmHg)	82.1 ± 14.9	86.8 ± 18.0	79.8 ± 12.8	0.178
Antihypertensive drug use (*n*; %)	14; 37.8	3; 25.0	11; 44.0	0.306
Lipid profile				
Cholesterol (mg/dL)	405.0 ± 153.3	380.6 ± 116.1	415.7 ± 168.2	0.535
Triglyceride (mg/dL)	188.3 ± 122.6	197.8 ± 83.6	183.4 ± 126.6	0.726
Low density lipoprotein-cholesterol (mg/dL)	290.6 ± 123.8	258.7 ± 104.7	304.5 ± 131.9	0.427
High density lipoprotein-cholesterol (mg/dL)	82.9 ± 39.6	54 ± 25.2	92.6 ± 39.8	0.152
Statin use (*n*; %)	22; 59.5	7; 58.3	15; 60.0	1.000
Laboratory data at biopsy				
Serum Creatinine (mg/dL)	1.0 ± 0.6	0.8 ± 0.2	1.0 ± 0.7	0.250
eGFR (mL/min/1.73m^2^)	97.8 ± 29.9	107.1 ± 20.4	93.4 ± 33.0	0.195
Serum Albumin (g/dL)	2.2 ± 0.9	2.4 ± 1.1	2.2 ± 0.8	0.507
UPCR (g/g)	8.2 ± 4.9	7.3 ± 4.3	8.6 ± 5.2	0.465
Urine RBC (/HPF)	8.4 ± 16.4	12.3 ± 27.8	6.4 ± 6.2	0.482
Serum IgM (mg/dL)	164.8 ± 117.7	201.9 ± 171.9	147.0 ± 78.8	0.188
Serum IgM/IgG (%)	36.6 ± 52.7	50.6 ± 88.6	29.8 ± 20.7	0.267

*p < 0.05. Values are expressed as mean ± standard deviation. *Abbreviations*: *IgM* Immunoglobulin M, *eGFR* Estimated glomerular filtration rate, *UPCR* Urine protein to creatinine ratio, *RBC* Red blood cells, *HPF* High power field

### Immunosuppressive agent regimens

A summary of immunosuppressive treatment is listed in Supplementary Table 1. Two patients (both the negative IgM group) have been follow-up for only 1 year, and others have been follow-up for more than 2 years. The average follow-up duration is 23.5 months. They all received immunosuppressive therapy after the biopsy. Prednisolone is the first-line immunosuppressant in this study. Only two patients received parenteral pulse methylprednisolone therapy (one patient belongs to the negative IgM group; another is in the positive IgM group). Three patients never used steroids (one patient received CsA therapy in the positive IgM group; one patient received CsA in the negative IgM group; another patient received CYC in the negative IgM group). The dosage of immunosuppressive treatment was not significantly different between the two groups.

### Treatment responses

Response to therapy is presented in Table 2. The time to partial remission, complete remission, and first relapse after treatment were not significantly different between the two groups. Five patients did not achieve complete remission (three patients in the negative IgM group, 12.0%; two patients in the positive IgM group, 16.7%, *p* = 1.000). The frequency of relapses was significantly different between these two groups (0.25 ± 0.37 episodes/year in the negative IgM group vs. 0.75 ± 0.59 episodes/year in the positive IgM group, *p* = 0.029) during 2 years.

**Table 2:** The treatment response in adult-onset minimal change disease on light microscopy

Parameters	All	IgM (+)	IgM (−)	*p*-Value
Patient number (*n*)	37	12	25	
Time to partial remission after treatment (days)	34.0 ± 75.0	51.8 ± 125.9	25.4 ± 30.7	0.487
Time to complete remission after treatment or last follow-up (days)	152.2 ± 230.9	154.7 ± 269.9	151.0 ± 215.8	0.965
No complete remission during two years (*n*; %)	5; 13.5	2; 16.7	3; 12.0	1.000
Time to first relapse after treatment (days)	357.1 ± 179.8	347.6 ± 167.8	365.4 ± 200.9	0.856
Frequency of relapses during two years (episodes/year)	0.41 ± 0.50	0.75 ± 0.59	0.25 ± 0.37	0.029*

*p < 0.05. Values are expressed as mean ± standard deviation. *Abbreviation*: *IgM* Immunoglobulin M

Univariate and multivariate linear regression analysis of the frequency of relapses were presented in Tables 3 and 4. Two factors (IgM deposition in IF microscopy and prednisolone daily dose/body weight during 2 years) were associated with frequency of relapses (IgM deposition, regression coefficient B 0.464, 95% CI 0.146–0.781, *p* = 0.006; and prednisolone daily dose, regression coefficient B 1.660, 95% CI 0.07–3.25, *p* = 0.041) by univariate analysis. Further multivariate linear regression analysis disclosed that only IgM deposition was independently associated with the frequency of relapses (regression coefficient B 0.450, 95% CI 0.116–0.784, *p* = 0.010).

**Table 3:** Univariate linear regression analysis of the frequency of relapses in adult-onset minimal change disease on light microscopy

Parameters	Coefficient B	95% CI	*p*-Value
IgM deposition on immunofluorescence microscopy	0.464	0.146–0.781	0.006*
Serum IgM	0.001	0.000–0.002	0.179
Serum IgM/IgG	0.002	−0.001-0.005	0.129
Prednisolone daily dose/body weight during two years	1.660	0.070–3.250	0.041*
Cyclosporine daily dose/body weight during two years	0.077	−0.456-0.611	0.750
Mycophenolic acid daily dose/body weight during two years	−0.075	−0.216-0.067	0.191
Cyclophosphamide daily dose/body weight during two years	0.475	−2.775–3.724	0.594
*p < 0.05. *Abbreviations*: *IgM* Immunoglobulin M, *CI* Confidence interval.

**Table 4:** Multivariate linear regression analysis of the frequency of relapses in adult-onset minimal change disease on light microscopy

Parameters	Coefficient B	95% CI	*p*-Value
IgM deposition on immunofluorescence microscopy	0.450	0.116–0.784	0.010*
Prednisolone therapy daily dose/body weight during two years			0.060

*p < 0.05. *Abbreviations*: *IgM* Immunoglobulin M, *CI* Confidence interval

### Subgroup analysis

We divided patients into two subgroups according to mean (Mean age was 40 years; mean eGFR was 97.8 mL/min/1.73m^2^; mean cholesterol level was 405 mg/dL) and clinical definition (the presence of nephrotic-range proteinuria; the presence of microscopic hematuria). Figure 2 showed the change in the frequency of relapses in different subgroups by mean and one standard deviation (SD). The reference line (0.905 episodes/year) in Fig. 2 was the mean value plus one SD of the frequency of relapses for all patients. It demonstrated that some subgroups crossed the reference line, including older age group (Age ≧ 40 years), male group, lower eGFR group (eGFR < 97.8 mL/min/1.73m^2^), hypercholesterolemia group (cholesterol ≧ 405 mg/dL), hypoalbuminemia group (serum albumin < 2.5 g/dL), proteinuria groups (both UPCR < 3.5 g/g and UPCR ≧ 3.5 g/g), and no microscopic hematuria group (urine RBC < 3 /HPF).

## Discussion

Several pediatric studies examined the clinical symptoms, steroid response, relapses, and renal outcomes in idiopathic nephrotic syndrome children with or without IgM mesangial deposition. We, to the best of our knowledge, are the first to investigate the predictive value of IgM mesangial deposition on patient outcomes in newly-diagnosed MCD adults. IgM nephropathy (IgMN) is defined by its immunohistologic features as IgA nephropathy, i.e., the diffuse mesangial deposition of IgM [14]. In our study, the IgM was not deposited diffusely, but all the IgM depositions were distributed exactly at the mesangial region in all the 12 patients upon IF examination. Therefore, we did not name these patients as IgMN, but the term positive IgM mesangial deposition was used instead. In our adult-onset MCD cohort, the multivariate linear regression analysis revealed that IgM deposit positivity is independently associated with a significantly higher frequency of relapses. Besides, further subgroup analysis showed that patients who developed MCD at the age of > 40 years old were associated with a higher frequency of relapses than those < 40 years old.

Previous studies showed a significantly higher mean serum IgM level in children with positive IgM deposition [15, 16]. In our study, there was a trend of increased IgM and IgM/IgG ratio in the positive IgM group but did not achieve statistical significance. This was probably due to the small sample size. Previous studies showed the IgM deposition increased risks of chronic kidney disease (CKD) and end-stage kidney disease in children for more than 10 years of follow-up [2, 5], illustrating the clinical implication of IgM positivity in MCD. Our study did not examine renal failure due to the reserved eGFR and the relatively short follow-up period in our cohort, but further research of IgM positivity on long-term renal outcomes in adult-onset MCD is warranted.

Intriguingly, our study found the frequency of relapses was more common in men with IgM deposition by our subgroup analysis. In an MCD study composed of both children and adults, gender was not a determinant of renal function progression [4]. Nevertheless, it has been reported that the renal function of males declined more rapidly than females in nondiabetic CKD [17].

In previous studies, children with IgM deposition were more likely to have hypertension [2, 18] and hematuria [5, 18]. There was no difference in blood pressure, lipid profile, renal function, hematuria, and proteinuria in MCD with or without IgM deposition [1, 6, 19]. Our study yielded similar results. Besides, according to our subgroup analysis, a higher frequency of relapses was more common in patients with lower eGFR < 97.8 mL/min/1.73m^2^, serum albumin < 2.5 g/dL group, serum cholesterol ≧ 405 mg/dL, the presence of nephrotic-range proteinuria, and the absence of microscopic hematuria. In accordance with our study, a previous study also demonstrated that microscopic hematuria was a favorable sign [20].

There are no randomized controlled trials on the treatment of MCD with IgM deposition. Corticosteroids constitute the mainstay of therapy in MCD. The prevalence of steroid resistance in idiopathic nephrotic syndrome patients with positive IgM deposition was inconclusive, varying from 0 to 52% [14]. Several studies showed a higher steroid dependence in MCD children with IgM deposition [2, 9]. Conversely, IgM deposition was not related to increased steroid resistance and steroid dependence in other pediatric studies [1, 18]. In our adult MCD cohort, there was also no difference between steroid resistance between patients with or without IgM deposition. Furthermore, in terms of time to partial remission, time to complete remission, and time to the first relapse after treatment, there was no difference between these two groups.

Adjuvant immunosuppressive therapy includes CsA, CYC, MPA, and levamisole, etc. [21]. Several studies evaluated the effect of adjuvant immunosuppressive therapy in idiopathic nephrotic syndrome [22–24]. CsA and CYC significantly reduced relapse risk compared to prednisolone alone in the frequent relapsing idiopathic nephrotic syndrome in pediatric patients [22]. In a study of idiopathic nephrotic syndrome, CsA and CYC are both effective and well-tolerated in adults and children [23]. CYC has more side effects, such as bone marrow suppression, gonadal toxicity, infection, seizure, malignancies, etc. [24]. Therefore, using CsA as first-line therapy in steroid-resistant nephrotic syndrome was indicated [25]. The patients with positive IgM deposition had a better response to CsA [3], and when combined with prednisolone, it can be more effective than prednisolone alone in MCD children with positive IgM deposition [26]. No significant difference in response to non-corticosteroid treatment was found between the MCD patients with or without IgM positivity [1, 2, 18, 27]. Our sample size was too small to evaluate the effect of adjuvant immunosuppressive therapy.

There were some limitations to this study. First, the optimal evaluation of the subgroup analysis was hindered due to the small sample size. Second, the follow-up period was relatively short, meaning further evaluation of renal function was halted. Third, constraints of retrospective study apply, indicating that the decision on dosages and types of immunosuppressive agents was at the discretion of each attending nephrologist. However, it should be noted that the average dosages of immunosuppressive agents were similar between patients with and without IgM mesangial deposition, as shown in Supplementary Table 1.

## Conclusions

Our findings indicated that newly diagnosed MCD adults with IgM mesangial deposition were more likely to experience disease relapses than those without. This effect was even more apparent in patients with older age, male, hypoalbuminemia, lower eGFR, presence of nephrotic-range proteinuria, or the absence of microscopic hematuria. Previous pediatric studies recommended that for this particular group of patients, the immunosuppressive agents may be tapered slowly with close follow-up or might be benefited from combined immunosuppressive therapy than prednisolone alone [22, 23, 26]. Therefore, we suggest intensive monitoring of disease activity in MCD adults with a positive IgM mesangial deposition. Whether these patients may benefit from a prolonged or combined immunosuppressant treatment deserves further investigation.

## Supplementary Information

**Additional file 1: Supplementary Table 1**. Dosage of immunosuppressive agents in adult-onset minimal change disease.

## Data Availability

All data generated or analyzed during this study are included in this published article and its supplementary information file.
