# Supplementary material for: IgM mesangial deposition as a risk factor for relapses of adult-onset minimal change disease
Source: BMC Nephrol. 2021 Jan 12;22:25. doi: 10.1186/s12882-021-02234-z (PMC7802152; doi:10.1186/s12882-021-02234-z)
Supplement: Supplementary file 1 — Additional file 1: Supplementary Table 1. Dosage of immunosuppressive agents in adult-onset minimal change disease. [file 12882_2021_2234_MOESM1_ESM.docx]

**SUPPLEMENTARY MATERIAL**

| **Supplementary Table 1**. Dosage of immunosuppressive agents in adult-onset minimal change disease on light microscopy. | | | | | |
| --- | --- | --- | --- | --- | --- |
| Parameters | | All | IgM (+) | IgM (-) | *p*-Value |
| Patient number (*n*) | | 37 | 12 | 25 |  |
| Prednisolone (*n*; %) | | 34; 91.9 | 11; 91.7 | 23; 92.0 |  |
|  | Daily dose/body weight (mg/kg/day) during two years | 0.26 ± 0.14 | 0.28 ± 0.15 | 0.25 ± 0.13 | 0.617 |
|  | Daily dose/body weight (mg/kg/day) in first year | 0.31 ± 0.16 | 0.30 ± 0.10 | 0.31 ± 0.18 | 0.752 |
|  | Daily dose/body weight (mg/kg/day) in second year | 0.22 ± 0.23 | 0.27 ± 0.31 | 0.18 ± 0.16 | 0.322 |
| Cyclosporine (*n*; %) | | 16; 43.2 | 5; 41.7 | 11; 44.0 |  |
|  | Daily dose/body weight (mg/kg/day) during two years | 2.31 ± 0.78 | 2.02 ± 0.72 | 2.45 ± 0.79 | 0.316 |
|  | Daily dose/body weight (mg/kg/day) in first year | 2.43 ± 0.99 | 2.43 ± 0.99 | 2.44 ± 1.07 | 0.994 |
|  | Daily dose/body weight (mg/kg/day) in second year | 2.03 ± 0.74 | 1.66 ± 0.74 | 2.17 ± 0.73 | 0.256 |
| Mycophenolic acid (*n*; %) | | 6; 16.2 | 1; 8.3 | 5; 20.0 |  |
|  | Daily dose/body weight (mg/kg/day) during two years | 8.56 ± 3.73 | 9.48 | 8.37 ± 4.14 | 0.820 |
|  | Daily dose/body weight (mg/kg/day) in first year | 9.51 ± 3.58 | 10.19 | 9.28 ± 4.34 | 0.873 |
|  | Daily dose/body weight (mg/kg/day) in second year | 6.79 ± 3.10 | 5.18 | 7.33 ± 3.56 | 0.653 |
| Cyclophosphamide (*n*; %) | | 5; 13.5 | 1; 8.3 | 4; 16.0 |  |
|  | Daily dose/body weight (mg/kg/day) during two years | 1.71 ± 0.40 | 1.91 | 1.66 ± 0.44 | 0.651 |
|  | Daily dose/body weight (mg/kg/day) in first year | 1.65 ± 0.43 | 1.91 | 1.56 ± 0.48 | 0.594 |
|  | Daily dose/body weight (mg/kg/day) in second year | 0 | 0 | 0 |  |
| Values are expressed as mean ± standard deviation. Abbreviation: IgM, immunoglobulin M. | | | | | |
